# Exploring definitions of porcine respiratory disease complex in the literature: a scoping review protocol

**DOI:** 10.3389/fvets.2026.1765099

**Published:** 2026-03-30

**Authors:** Beatriz Garcia-Morante, Maria Rodrigues da Costa, Joaquim Segalés

**Affiliations:** 1 IRTA, Animal Health, Centre de Recerca en Sanitat Animal (CReSA), Campus de la Universitat Autònoma de Barcelona (UAB), Bellaterra, Catalonia, Spain; 2 Unitat Mixta d’investigació IRTA-UAB en Sanitat Animal, Centre de Recerca en Sanitat Animal (CReSA), Campus de la Universitat Autònoma de Barcelona (UAB), Bellaterra, Catalonia, Spain; 3 WOAH Collaborating Centre for the Research and Control of Emerging and Re-emerging Swine Diseases in Europe (IRTA-CReSA), Bellaterra, Barcelona, Spain; 4 Centre for Epidemiology and Planetary Health, School of Veterinary Medicine and Biosciences, SRUC (Scotland’s Rural College), Inverness, United Kingdom; 5 Departament de Sanitat i Anatomia Animals, Facultat de Veterinària, Universitat Autònoma de Barcelona (UAB), Campus de la UAB, Bellaterra, Spain

**Keywords:** case definition, co-infection, pig, porcine respiratory disease complex, PRDC, respiratory disease, scoping review, swine

## Abstract

**Background:**

Porcine respiratory disease complex (PRDC) is a major health and economic concern in commercial swine production, contributing to reduced growth performance, increased mortality, elevated treatment costs, and widespread antimicrobial use. Although the term and its acronym are widely used in both research and clinical practice, its definition varies considerably across studies. Definitions may rely solely on clinical signs or incorporate combinations of pathological findings, laboratory diagnostics, performance indicators, and environmental or management factors. This lack of definition consistency challenges disease surveillance, diagnosis, and comparability across studies, potentially hindering the development of effective interventions. Scoping reviews are particularly suited for clarifying how complex terms are defined, mapping research approaches, and identifying gaps in knowledge. The objective of this scoping review is to systematically map and describe how PRDC has been defined and operationalized in the scientific literature.

**Methods and analysis:**

This review will follow a five-step refined methodological framework guided by the Joanna Briggs Institute. Eligible sources will include peer-reviewed and grey literature published in English language from 1990 onwards. Searches will be conducted in PubMed/MEDLINE, Web of Science, CAB Abstracts, and Scopus, supplemented by grey literature sources such as OpenGrey, ProQuest, and Google Scholar. Additionally, all open-access issues of the Journal of Swine Health and Production will be manually reviewed. Two reviewers will independently screen titles, abstracts, and full texts, with disagreements resolved by consensus or a third reviewer. Data will be extracted on study characteristics, PRDC definitions, diagnostic criteria, pathogens considered, and production context, including environmental and/or housing/management conditions. Results will be reported in accordance with PRISMA-ScR, using descriptive tables and narrative synthesis to explore variability and consistency in PRDC definitions.

**Discussion:**

This protocol outlines a scoping review designed to chart how PRDC has been defined in swine health research. By identifying areas of consensus and divergence, the review will support the development of more consistent case definitions, enhance cross-study comparability, and inform future research and systematic reviews.

## 1 Introduction

Porcine respiratory disease complex (PRDC) is one of the most important and costly health problems in commercial swine production worldwide (1). It is associated with reduced growth performance, poor feed efficiency, and increased mortality and treatment costs (2, 3), as well as being a major driver of therapeutic and metaphylactic antimicrobial use in pigs (4). In addition to its economic burden, PRDC raises significant animal welfare concerns, since it may be linked to prolonged clinical signs such as coughing, labored breathing, lethargy, and fever (2, 3, 5).

PRDC is not a single disease; rather, it is considered a multifactorial respiratory condition resulting from interactions between bacterial and viral pathogens, host factors, environmental conditions, and management practices (2, 3, 5, 6). The term gained widespread use in the 1990s as a practical way to describe cases of pneumonia of mixed etiology, particularly during the nursery and grower–finisher phases of pig production (2). Pathogens such as *Mycoplasma hyopneumoniae*, *Pasteurella multocida*, *Actinobacillus pleuropneumoniae*, porcine reproductive and respiratory syndrome virus (PRRSV), porcine circovirus 2 (PCV2), and swine influenza virus (swIAV) are frequently detected in PRDC cases (7).

Although PRDC is widely recognized in veterinary literature, its precise definition appears to vary. Some reports describe PRDC primarily in terms of clinical signs, while others incorporate pathological, microbiological, or molecular findings, sometimes together with production indicators such as average daily weight gain or mortality (8, 9). In addition, many details of the involvement of environmental factors in swine respiratory diseases are still not fully understood (6). Lastly, while PRDC is fundamentally a polymicrobial process, substantial variation exists across studies in how the presence and contribution of multiple pathogens are documented, which directly contributes to inconsistencies in PRDC definitions. This variability in the pathogens considered, and in the evidence required for their involvement, contributes directly to heterogeneity in PRDC definitions across the literature. The extent, consistency, and implications of these different definitions have not yet been systematically examined. This lack of clarity may hinder comparability between studies, complicate surveillance and diagnosis, and influence treatment and control strategies.

Scoping review methodology provides a systematic way to map the breadth and depth of research in areas where concepts are variably defined and operationalized. Scoping reviews are particularly useful when the literature is heterogeneous, when terminology is unclear, or when the purpose is to clarify definitions and identify knowledge gaps (10, 11). Unlike systematic reviews, which focus on answering narrowly defined questions and synthesizing evidence of effectiveness, scoping reviews aim to describe how a topic has been studied, the range of methods employed, and the key concepts that underpin it (12). In veterinary medicine, scoping reviews have recently been applied to conditions such as calf respiratory disease (13) and tail-biting in pigs (14), highlighting inconsistencies in case definitions and research priorities.

The objective of this protocol is therefore to outline the planned methods for a scoping review that will systematically map the literature on PRDC, with a particular focus on its case definitions and diagnostic criteria. Specifically, the review will aim to describe how PRDC has been defined across study designs, production systems, and geographic regions; chart the components most frequently included in those definitions; and identify areas where definitions converge or diverge. By doing so, the planned review will provide a comprehensive overview of current practice and lay the groundwork for developing clearer and more consistent definitions of PRDC in future research and practice.

## 2 Methods

The methodological approach of this study is based on the scoping review framework. This approach is particularly suited to mapping how PRDC has been defined and operationalized in scientific literature, and to identify areas of convergence, divergence, and knowledge gaps. The protocol has been registered with the Open Science Framework^1^ and is reported in accordance with the Preferred Reporting Items for Systematic Reviews and Meta-Analyses Protocols (PRISMA-P) statement (15, 16) (see checklist in Supplementary File 1). The review follows the foundational framework proposed by Arksey and O’Malley (10), subsequently refined by Levac et al. (11) and further developed in the Joanna Briggs Institute (JBI) Reviewer’s Manual (17). Reporting will adhere to the PRISMA Extension for Scoping Reviews (PRISMA-ScR) checklist (12).

The review protocol will proceed through the following stages: (i) identifying the research questions and objectives; (ii) locating relevant published and grey literature; (iii) selecting studies based on predefined eligibility criteria; (iv) extracting and charting data; and (v) collating, summarizing, and reporting the results.

### 2.1 Hypothesis, research questions, and outcomes

#### 2.1.1 Hypothesis

We hypothesize that definitions of PRDC vary substantially across the scientific literature, reflecting differences in geographic regions, pig populations, production systems, and diagnostic approaches. We further hypothesize that PRDC definitions differ in the relative emphasis placed on clinical signs, pathological findings, laboratory diagnostics (i.e., pathogen detection), performance indicators, and environmental or management factors.

#### 2.1.2 Research questions and outcomes

The overarching aim of this scoping review is to systematically map how PRDC has been defined in the literature. This includes examining the (a) conceptual (what PRDC is considered to be; e.g., multifactorial, syndromic, co-infection-driven), (b) diagnostic (how PRDC is identified; e.g., clinical signs, pathology, laboratory testing, performance indicators), and (c) contextual (where/when PRDC is defined; e.g., production stage, housing/management, environmental exposures) elements that constitute PRDC case definitions in the literature. To achieve this, the review will be guided by the following research questions, which also define the outcomes to be extracted and synthesized:

- Primary outcome/research question: How has PRDC been defined and operationalized across studies? This includes the specific components of the definitions (e.g., clinical signs, pathological findings, diagnostic tests, performance indicators, environmental or housing/management conditions).
- Secondary outcomes/research questions: (i) Which pathogens are most frequently included in PRDC definitions? and (ii) How do PRDC definitions vary across study designs, pig populations, production systems, and geographic regions?

These outcomes/questions have been prioritized to address the central objective of identifying areas of consensus, divergence, and gaps in the conceptualization of PRDC.

### 2.2 Identification of relevant literature

#### 2.2.1 Eligibility criteria

Studies will be eligible for inclusion if they are published in English language from January 1990 to date, reflecting the period when PRDC terminology was first conceived in the veterinary literature (2). Eligible studies must involve pigs (*Sus scrofa domesticus*), encompassing all pig population types, including nursery pigs, grower–finisher pigs, breeding herds (sows, boars, and gilts), mixed-age populations, minipig breeds used in research settings (e.g., Göttingen or Yucatan), and non-commercial pig populations such as pet or show pigs. Studies conducted under any production or management system are eligible, including commercial production systems as well as experimental, laboratory, or other non-commercial settings. No restrictions are applied to geographic region, and studies from all countries and production intensities worldwide are eligible for inclusion. Both field-based and laboratory or experimental studies will be considered eligible, provided that the term “porcine respiratory disease complex” or its acronym PRDC is explicitly stated and accompanied by a definition, case description, or diagnostic criteria. Accepted publication types include original research articles, reviews, commentaries, short communications, books and book chapters, and editorials. Selected grey literature will also be considered, specifically doctoral or master’s theses, government or agency reports, technical reports from research institutes, and documents produced by international organizations (e.g., WOAH, FAO, EFSA).

Studies will be excluded if they mention PRDC without providing any case definition, description, or criteria, or if they focus exclusively on the pathogenesis of individual pathogens without reference to PRDC as a complex. Excluded publication types include conference proceedings, book reviews, wiki articles, blogs, websites, practice guidelines, leaflets and brochures, policy statements, reports on scientific meetings, and corporate literature or data.

#### 2.2.2 Information sources

Primary databases for the literature search will include PubMed/MEDLINE, Web of Science Core Collection, Scopus, and CAB Abstracts. Secondary sources for grey or difficult-to-locate literature will include Google (Google Scholar and Google Books), OpenGrey, ProQuest Dissertations & Theses, and WorldWideScience.org. To ensure comprehensive coverage, all open-access issues of the Journal of Swine Health and Production (JSHAP) will be manually reviewed, as this peer-reviewed journal is not indexed in major bibliographic databases. Additionally, hand searches of reference lists from included studies, reviews, and other relevant documents will be conducted to identify further publications not directly captured in the database searches [snowballing; (18)].

#### 2.2.3 Search strategy

A comprehensive search strategy will be developed using a combination of controlled vocabulary (e.g., MeSH terms) and free-text keywords related to “porcine respiratory disease complex” and “PRDC.” The strategy will aim to identify both published and unpublished studies, following the three-step approach recommended in scoping review methodology (17):

1. Initial limited search: At the time of protocol registration with the OSF (August 2025), an exploratory search of MEDLINE (via PubMed) was conducted to identify relevant articles. Text words contained in the titles and abstracts of these articles, and the index terms used to describe them, were analyzed to inform the development of the full search strategy. A preliminary draft search string for PubMed/MEDLINE is provided in Supplementary File 2.
2. Database-specific searches: Tailored strategies will then be developed and executed for each database and grey literature source listed above.
3. Citation searching: The reference lists of all included studies and relevant reviews will be screened to identify additional eligible records [snowballing; (18)].

Whenever possible, citations will be retrieved directly into Rayyan QCRI (RRID: SCR_017584), a web-based tool designed to facilitate systematic review screening by allowing independent, blinded screening by multiple reviewers and efficient resolution of conflicts. Only sources not directly compatible with Rayyan (e.g., grey literature or manually searched journals) will first be exported into EndNote (RRID: SCR_014001) for deduplication before being uploaded into Rayyan for screening. At the time of protocol submission, no finalized search string is provided. Instead, a preliminary search strategy and list of keywords have been developed and pilot tested to identify relevant literature, as reported in Supplementary File 2. This strategy was intentionally broad and will be refined during pilot testing and fully reported in the final review, in accordance with PRISMA-ScR guidelines (12).

### 2.3 Study selection

A two-step screening process will be employed. First, titles and abstracts will be screened independently by two reviewers to assess eligibility against the predefined inclusion and exclusion criteria described above. Documents not meeting the predefined criteria will be excluded from full-text review. Second, full-text articles of potentially eligible studies will be independently assessed by the same two reviewers using the same criteria applied in greater depth at full text. In cases of uncertainty or disagreement, a third reviewer will re-evaluate the study, and final decisions will be made through discussion and consensus. Prior to commencing full screening, a pilot test of the eligibility criteria will be conducted by the three reviewers on a small subset of records to calibrate reviewer judgments and, if necessary, refine operational wording of criteria without altering their substantive intent. The screening process and the number of included and excluded studies will be documented using a PRISMA-ScR flow diagram (12).

### 2.4 Data extraction and charting

Data will be extracted from papers included in the scoping review by two independent reviewers using a standardized charting form adapted from the JBI template (17) and developed by the reviewers (Supplementary File 3). Extracted variables will include bibliographic details (author, year, country), study design, production stage and system, PRDC definition criteria (e.g., clinical, pathological, microbiological, molecular, performance-based, environmental), and pathogens considered including prevalence if reported. Before data extraction, the extraction tool will be piloted with sources of different designs (e.g., qualitative, randomized controlled trials) to ensure consistency between reviewers, as per guidance in the JBI Manual (17). Any necessary adjustments to the charting categories will be made through discussion within the review team before full data extraction begins. Then, two reviewers will independently chart the data, with regular meetings to resolve discrepancies and refine categories. Any disagreements between reviewers will be resolved with the additional reviewer. Hence, the charting process will be iterative, allowing for the modification of the draft data extraction tool as necessary. Any modifications will be detailed in the scoping review. If appropriate, authors of papers will be contacted a maximum of two times to request missing or additional data, where required. Authors will be contacted a maximum of two times, with a minimum interval of 2 weeks between attempts. If no response is received after these attempts and the missing information is critical for inclusion, the source will be excluded from the review.

### 2.5 Collating, summarizing, and reporting results

The review process will be documented using a PRISMA-ScR flow diagram (12). Following study selection in Rayyan, data from included studies will be collated and charted using Microsoft Excel. Descriptive characteristics (e.g., study type, method, location) will be analyzed empirically, while findings related to PRDC definitions and conceptual frameworks will be synthesized narratively. When applicable, quantitative summaries will include frequency tables, graphs (e.g., bubble plots), and study matrices. Thematic analysis will be used to identify and describe recurring patterns, gaps, and emerging themes or topics that may be gaining attention in the literature but have not yet been consistently defined. Content analysis methods will support the classification of topics into coherent and clearly defined themes. Two reviewers will conduct the thematic synthesis independently, with results consolidated through consensus.

## 3 Discussion

The idea for this scoping review was motivated by recent work by Churchill et al. (13), who conducted a synthesis of case definitions for respiratory disease in dairy calves. Their findings revealed substantial variability in how syndromic terms are defined and applied across studies, raising concerns about diagnostic consistency, comparability, and implications for surveillance and intervention strategies. Recognizing similar challenges in swine health research, particularly regarding a complex condition like PRDC (2, 3, 5), we identified the interest to systematically examine how PRDC has been defined in the scientific literature. Although PRDC is widely used in swine health research and practice, the absence of a standardized definition contributes to variability across studies and may also affect diagnostic consistency and treatment decisions, among others. In this context, a scoping review is particularly well-suited to clarify how PRDC has been defined and operationalized, highlighting consistencies and divergences and laying the groundwork for more harmonized case definitions.

The protocol draws on established frameworks for scoping reviews (10, 11) and follows the PRISMA-ScR guidelines to ensure methodological transparency and rigor (12). It also aligns with recommendations for applying scoping reviews in veterinary medicine (19). By including both peer-reviewed and grey literature, and through independent screening and data extraction by two reviewers, the protocol is designed to enhance comprehensiveness and reduce the risk of selection bias. Consistent with the scoping review methodology, no formal appraisal of study quality or risk of bias will be undertaken, as the objective is to map definitions rather than evaluate intervention effects or the internal validity of individual studies.

Some limitations are anticipated. Restricting the search to English language publications may result in the omission of relevant studies published in other languages, particularly given the global nature of swine production. However, the review was limited to studies published in English to ensure consistency in terminology and feasibility of screening. Additionally, variability in the level of detail provided in primary studies may constrain the ability to extract comparable information across sources. The decision to limit the search to publications from 1990 onwards reflects the emergence of the term PRDC in the literature (2), although earlier studies of multifactorial respiratory disease in pigs not labeled under this term may not be captured.

Despite these limitations, the review is expected to provide a valuable foundation for improving conceptual clarity in the field of swine respiratory health. By complementing broader initiatives to strengthen case definition development in animal health (20), findings will offer an evidence-based overview on how PRDC has been defined to date. While a single, globally applicable consensus definition of PRDC is likely challenged by differences in pathogen ecology, production systems, and epidemiological contexts across regions, this scoping review does not seek to impose uniformity. Instead, it aims to systematically document and compare existing definitions, making areas of convergence and divergence explicit. This approach will support more transparent, context-aware interpretation of the literature and provide a solid starting point for informed discussion on whether, and to what extent, harmonization of PRDC case definitions is feasible or desirable. In doing so, the findings are expected to inform the design of subsequent systematic reviews or primary research.

As the review involves only the analysis of publicly available literature, it does not require ethics approval. At the time of submission, study screening and final data extraction had not yet commenced. Eligibility criteria have been predefined, and a draft data charting form has been developed and will be piloted and refined iteratively prior to full data extraction, in accordance with JBI guidance (17). The protocol presented here provides a detailed account of the study’s rationale and methodology. Any deviations from the protocol will be reported and justified in the appropriate section of the methods in the final review, and copyright and intellectual property rights will be respected throughout. The results will be disseminated through an article published in a peer-reviewed journal, in addition to presentations at conferences and scientific events.

## Data Availability

The original contributions presented in the study are included in the article/Supplementary material, further inquiries can be directed to the corresponding author.

## Footnotes

1 OSF registration: https://osf.io/mt3qk.
