# Supplementary material for: Exploring definitions of porcine respiratory disease complex in the literature: a scoping review protocol
Source: Front Vet Sci. 2026 Mar 30;13:1765099. doi: 10.3389/fvets.2026.1765099 (PMC13072483; doi:10.3389/fvets.2026.1765099)
Supplement: Supplementary file 2 [file Supplementary_file_2.docx]

Supplementary Material

# Search strategy

An initial limited search of MEDLINE (via PubMed) was conducted during August 2025 to identify relevant articles related to porcine respiratory disease complex (PRDC). Text words contained in the titles and abstracts of relevant articles, and the index terms used to describe the articles will be used to inform the development of the full search strategy.

Database/Platform: MEDLINE (via PubMed)

Date of search: August 2025

Limits applied: English language, from January 1990 to present

Preliminary Search String (PubMed):

("Porcine Respiratory Disease Complex"[Title/Abstract]

OR "PRDC"[Title/Abstract]

OR ("porcine"[Title/Abstract] AND "respiratory disease"[Title/Abstract])

OR ("swine"[Title/Abstract] AND "respiratory disease"[Title/Abstract]))

OR ("pig"[Title/Abstract] AND "respiratory disease"[Title/Abstract]))

OR ("pigs"[Title/Abstract] AND "respiratory disease"[Title/Abstract]))

OR ("porcine"[Title/Abstract] AND "pneumonia"[Title/Abstract])

OR ("swine"[Title/Abstract] AND "pneumonia"[Title/Abstract]))

OR ("pig"[Title/Abstract] AND "pneumonia"[Title/Abstract]))

OR ("pigs"[Title/Abstract] AND "pneumonia"[Title/Abstract]))

Results: The preliminary search retrieved 2,787 records. This preliminary string was intentionally broad to capture vocabulary and indexing practices. Relevant text words and index terms are basically “porcine respiratory disease complex" and "PRDC", which will be used to refine the comprehensive search strategy for PubMed, additional databases (Web of Science, Scopus, CAB Abstracts) and grey literature sources.
