# Supplementary material for: Exploring definitions of porcine respiratory disease complex in the literature: a scoping review protocol
Source: Front Vet Sci. 2026 Mar 30;13:1765099. doi: 10.3389/fvets.2026.1765099 (PMC13072483; doi:10.3389/fvets.2026.1765099)
Supplement: Supplementary file 3 [file Supplementary_file_3.docx]

Supplementary Material

# Draft data extraction charting form

(adapted from Peters et al., 2020, JBI Manual for Evidence Synthesis)

| **Category** | **Data item** | **Description / guidance for extraction** |
| --- | --- | --- |
| **Bibliographic details** | Author(s) | First author surname et al. |
|  | Year of publication | Year study/report was published |
|  | Title | Full title of the article/report |
|  | Country/region | Country(ies) where study was conducted or data collected |
|  | Source type | Journal article, thesis, report, book chapter, etc. |
| **Study characteristics** | Study design | Experimental, observational, case report, review, etc. |
|  | Pig population | Age category (nursery, grower, finisher, sow, mixed) and purpose (commercial, experimental, pet/show) |
|  | Production system | Intensive, extensive, experimental facility, etc. |
| **PRDC definition and criteria** | Reported definition of PRDC | Verbatim definition as described in the source |
|  | Criteria – clinical | Clinical signs included (e.g., coughing, dyspnoea, fever) |
|  | Criteria – pathological | Necropsy findings, gross lesions, histopathology |
|  | Criteria – laboratory | Microbiological culture, PCR, serology, sequencing |
|  | Criteria – performance-based | Production measures (mortality, ADG, feed efficiency, etc.) |
|  | Criteria –environmental | Housing, ventilation, temperature, stocking density, etc. |
|  | Criteria –management | Biosecurity, vaccination, treatment practices, feeding, all-in/all-out practices, etc. |
|  | Other criteria | Any additional factors (e.g., epidemiological, temporal) |
| **Pathogens considered** | Viral pathogens | List viruses (and prevalence if reported) explicitly associated with PRDC |
|  | Bacterial pathogens | List bacteria (and prevalence if reported) explicitly associated with PRDC |
|  | Other agents | Fungi, parasites, if applicable |
| **Additional information** | Key findings / notes | Any other relevant observations regarding PRDC definitions |
|  | Reviewer comments | Space for clarifications, uncertainties, or decisions during extraction |
